# What characterises women who eat potatoes? A cross-sectional study among 74,208 women in the Norwegian Women and Cancer cohort

**DOI:** 10.3402/fnr.v59.25703

**Published:** 2015-02-19

**Authors:** Lene A. Åsli, Tonje Braaten, Anja Olsen, Eiliv Lund, Guri Skeie

**Affiliations:** 1 Department of Community Medicine, University of Tromsø – The Arctic University of Norway, Tromsø, Norway; 2 Danish Cancer Society Research Center, Copenhagen, Denmark

**Keywords:** potato consumption, socioeconomic determinants, dietary factors, women's diet, nutrition, Norway, cross-sectional study, epidemiology

## Abstract

**Background:**

Studies of potato consumption have shown that age, region, socioeconomic status, and household structure are important determinants.

**Objective:**

This study aims to map which factors influence potato consumption among women in the Norwegian Women and Cancer (NOWAC) study.

**Design:**

A cross-sectional study using a postal questionnaire among 74,208 NOWAC participants aged 41–70.

**Results:**

Results showed that 56% of the women ate at least two potatoes a day. A north–south gradient in potato consumption was observed in logistic regression models (OR: 3.41, 95% CI: 3.19–3.64 for the north compared to the capital). Women in households with children had lower odds of high potato consumption than women living only with a partner, and women who lived alone had the lowest odds of all (OR: 0.39, 95% CI: 0.37–0.41). Smokers had higher odds of high potato consumption, while diabetics had lower odds. The odds of high potato consumption were greater among older women, and among those with lower income and education. In a sub-cohort, women who were dieting had lower odds of high potato consumption. Consumption of different foods varied in the low versus the high potato consumption group, with largest effect for fish and pasta/rice. The groups had similar nutrient densities.

**Conclusions:**

In addition to lifestyle and socioeconomic factors, health-related factors like smoking and diabetes were found to influence potato consumption. The high potato consumption group had an especially high consumption of fish and a low consumption of pasta/rice, though the nutrient density in the groups was similar.

The potato was introduced in Norway in the mid-1700s. Potato growing had its breakthrough during the war in 1807–1808, and it became an extremely important contributor in terms of food security and poverty alleviation (1). Today boiled potatoes, together with meat or fish, vegetables and gravy, are a central component of a Norwegian supper (2, 3) and are an important source of fibre, niacin, folate, and vitamin C (4, 5). Despite its role as a nutrient-rich staple food, there has been a drastic decline in the consumption of potatoes, particularly boiled potatoes, in Norway over the past 40 years (2, 6). This follows a similar trend that has been taking place in most Northern and Central European countries (2).

Potatoes have a high glycaemic index and load (7–9), which might be one reason why they have fallen into disrepute. Due to the dramatic effect potatoes have on blood sugar and insulin secretion, several researchers in nutrition have put potatoes on top of their food pyramid as a food that should be eaten only occasionally (10). Studies have shown that diets with low glycaemic index and/or low glycaemic load are associated with a reduced risk of several chronic diseases, such as type 2 diabetes, heart disease, and several cancers (11). Reviews of studies on potato consumption and human health have reported beneficial associations between potatoes and cardiometabolic health (5) and several cancers (12). However, studies on the long-term cancer-related health effects of potato consumption are lacking (12). There is also a lack of clinical trial data on the impact of potatoes on weight management, and the results of several cohort studies carried out on this topic are contradictory (5). Contradictory results have also been reported regarding potato consumption and diabetes (12). Potatoes are usually eaten as part of a meal, and therefore the impact potatoes have on disease risk may depend on the foods they are grouped with in a dietary pattern (5).

Previous studies have shown that age, area of residence, income, education and household structure are important determinants of potato consumption (2, 4, 13). The aim of this study is to gain a better understanding of which factors influence potato consumption among women in the Norwegian Women and Cancer (NOWAC) study, and which potential confounders should be considered in future studies of potato consumption and health outcomes.

## Experimental methods

### The NOWAC study

The NOWAC study is an ongoing, population-based prospective cohort study which started data collection in 1991. In total, the cohort consists of approximately 172,000 women, aged 30–70 years at recruitment. Women are randomly drawn from the Norwegian Central Population Register and sent an invitation letter, requesting informed consent to participate in the NOWAC study, and a comprehensive eight-page self-administered questionnaire.

The questionnaire includes questions on lifestyle, socioeconomic factors and health, as well as a semi-quantitative food-frequency questionnaire (FFQ) with 1–2 repeated measurements at 4- to 7-year intervals. The FFQ covers the habitual diet over the previous year. As new hypotheses have developed throughout the years, the questionnaire has continuously been improved, and questions have been included, omitted, or changed. The number of frequency questions on food, non-alcoholic and alcoholic drinks, and dietary supplements has therefore varied from 73 to 109 in the particular data sets from NOWAC included in the present study. The question on potato consumption has remained unchanged. Frequencies regarding consumption of different food items are asked as appropriate (per day, week, month, or year). Food items are either accompanied by questions on amounts consumed (in natural units, household units, or decilitres), or the questions are posed with a quantification (e.g. glasses of milk), or a standard portion (e.g. per potato). The weights of the foods and portions used are mostly derived from a Norwegian weights and measures table (14), and the daily intake of nutrients and energy is calculated using values from the Norwegian Food Composition Table (15, 16).

Further information on the food and nutrient calculation has been previously described (17), and a more detailed description of the NOWAC study has also been published previously (18). A description of the external validity of the NOWAC study is available elsewhere (19), along with a validation study (20), and a test–retest reproducibility study (21) of the FFQ. In addition, a validity study on self-reported physical activity (22) and diabetes (23) in the NOWAC study has been performed. The Regional Ethical Committee has approved the NOWAC study.

## Study sample

In the present study, 95,942 participants were preliminarily included. Baseline questionnaires were completed at recruitment by all participants during one of three periods: 1991–1992 (38,179 women), 1996–1998 (30,329 women), and 2003–2004 (27,434 women). Baseline data were used for all participants recruited in 1996–1998 and 2003–2004. For women recruited in 1991–1992, data from follow-up questionnaires completed in 1996–1998 were used. This decision was made as the follow-up questionnaire was more comprehensive than the baseline questionnaire these women completed in 1991–1992 and was also more comparable to the questionnaires completed by the rest of the participants. Only information on education was taken from the baseline questionnaire of women recruited in 1991–1992, as this information was not available in the follow-up questionnaire.

We excluded 1,022 women with missing information on potato consumption, and another 20,440 women due to the lack of information on the selected covariates (income, education, household structure, smoking, BMI, and physical activity) we used in the analysis. In addition, 270 women were excluded due to implausible daily energy intake (<2,500 kJ, >15,000 kJ), and two women were excluded due to implausible height. Hence, 74,208 women were finally included in the present analyses. We also performed analyses in a sub-cohort of 22,726 participants who answered questions on dieting.

## Statistical analysis

Data were analysed using STATA version 12 and SAS version 9.2. Frequency tabulation was performed, and Pearson's chi square test, Wilcoxon test, and linear regression analysis were used to test for significant differences between high and low potato consumption groups. Logistic regression analyses with 95% CIs and tests for linear trend across categories of age, income, and education were performed. For BMI and physical activity the test for trend was done on the medians in each category. The dependent variable in the logistic regression model was dichotomised as low and high potato consumption. Women who answered that they did not eat/seldom ate potatoes, ate 1–4 potatoes a week, 5–6 potatoes a week, or one potato per day were categorised as having low potato consumption. Those who answered that they ate two potatoes per day, three potatoes per day or ≥4 potatoes per day were categorised as having high potato consumption. Other variables examined were age (40–49, 50–59, and 60–70 years), area of residence (Oslo, east except Oslo, south, west, middle, north), income (<150,000; 151,000–300,000; 301,000–450,000; 451,000–600,000; 601,000–750,000; and >750,000 Norwegian krone), education (≤9, 10–12, and ≥13 years), smoking status (never, former, and current), body mass index (BMI: weight in kilograms divided by height in meters squared, <25: underweight/normal-weight, 25–29.9: overweight, ≥30: obese), diabetes (dichotomised), physical activity (1–3: low, 4–7: moderate, 8–10: high) (22), and dieting (dichotomised).

In order to determine whether there were children in the household, a variable called ‘household structure’ was generated by combining the existing variables; ‘number of persons in household’ and ‘marital status’. Women who were married/living with a partner, and reported no more than two people in the household were categorised as ‘living with partner’ (no children in household). Women who were single, widowed, or divorced and reported one person in the household were categorised as ‘living alone’, whereas those who reported at least two persons in the household were categorised as ‘living with children’.

Many women (12,875) did not answer the questions concerning diabetes, 1,293 women answered that they had diabetes, and 60,042 women answered they did not have diabetes. Due to results from a validation study of self-reported diabetes in the NOWAC study (23), we recoded those with missing information as ‘not having diabetes’. In the questionnaire, there was no distinction between the type 1 and type 2 diabetes. However, according to the validation study, the diabetes cases are mainly type 2 diabetes (89.4%) (23).

All food items are presented either as medians with 5th–95th percentile, or as mean consumption with age-adjusted means and age-adjusted nutrient density per energy intake (per 1,000 kJ). In the logistic regression model, food items were divided into appropriate portion sizes and used as continuous variables.

Due to the fact that we included groups of participants with at least 5 years between data collection, we adjusted all analyses by sub-cohort (data collection in 1996–1998 or 2003–2004). For non-dietary data, we present one model adjusted for age and sub-cohort, and one in which all the variables were mutually adjusted. For the question on dieting we also adjusted for energy intake. All the food items were adjusted for age and sub-cohort, in addition to a model where we also adjusted for energy intake. Stratification by sub-cohort, rather than adjustment for this variable, did not influence the estimates.

Usual regression diagnostics were performed to assess model fit. In addition, we tested for interactions between BMI and age, BMI and physical activity, and between age and energy intake. Also in the sub-cohort regarding women who answered questions on dieting, we tested for interactions between energy intake and several variables (age, BMI, and physical activity), in addition to interaction between age and BMI.

All *p*-values below 0.05 were considered statistically significant.

## Results

**Fig. 1:**
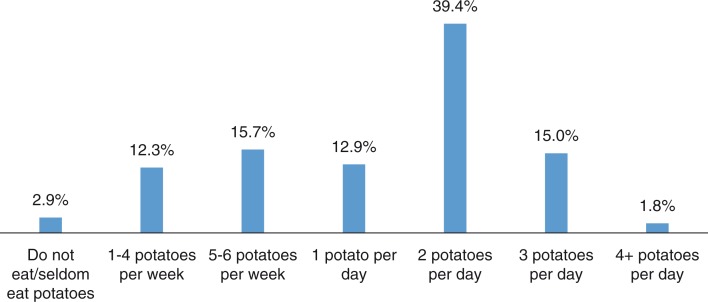
Frequency distribution of potato consumption: the NOWAC study. *n*=74,208.

Results showed that 56% of the women belonged to the high potato consumption group (ate at least two potatoes a day) (Fig. 1). The high potato consumption group consisted of more elderly women, women with lower income and education, more women living with a partner and more smokers (Table 1). For most lifestyle factors, the difference between the high and the low potato consumption group was highly significant (Table 1).

**Table 1:** Lifestyle characteristics of potato consumers in the NOWAC study

	Low (*n*=32,493)	High (*n*=41,715)	p*
Lifestyle, socioeconomic and health-related variables, %			
Age (years)			
40–49	44.5	39.4	
50–59	48.7	47.9	
60–70	6.8	12.7	<0.001
Area of residence			
Oslo	13.8	5.4	
East except Oslo	40.1	32.3	
South	4.9	4.6	
West	21.2	21.6	
Middle	7.2	8.3	
North	12.8	27.8	<0.001
Income (NOK)			
<150,000	5.9	9.2	
151,000–300,000	24.6	30.0	
301,000–450,000	24.9	30.8	
451,000–600,000	23.2	19.8	
601,000–750,000	13.6	7.8	
>750,000	7.7	2.4	<0.001
Education (years)			
≤9	15.2	29.6	
10–12	32.6	36.0	
≥13	52.2	34.4	<0.001
Household structure			
Living with partner	36.7	46.6	
Living alone	16.4	9.4	
Living with children	46.9	43.9	<0.001
Smoking status			
Never	35.0	35.4	
Former	39.1	32.5	
Current	25.9	32.0	<0.001
BMI			
Underweight/ normal-weight	62.6	59.4	
Overweight	28.3	31.1	
Obese	9.1	9.5	<0.001
Diabetes			
Diabetic	2.0	1.6	
Non-diabetic	98.0	98.4	<0.001
Physical activity			
Low	12.8	12.5	
Moderate	71.9	73.5	
High	15.3	14.0	0.012
Dieting†			
On diet	35.9	30.2	
Not on diet	64.1	69.8	<0.001

NOWAC, Norwegian Women and Cancer; NOK, Norwegian Kroner; Low, ≤1 potato per day; High, ≥2 potatoes per day

* *P*-value: Wilcoxon and chi square test

† Dieting; potato consumption in a sub-cohort (*n*=22,726).

Compared to the low potato consumption group, women in the high potato consumption group ate larger amounts of several foods, like fish, meat and bread, and also had a higher median consumption of nutrients like fat, carbohydrates, fibre, starch, niacin, vitamin C and folate (Table 2). In addition, there was a considerable difference in energy intake between the low and high potato consumption group, with the high consumption group having a higher median consumption (almost 700 kJ/day). For some foods, like pasta and rice, but also vegetables, median consumption was lower in the high potato consumption group. Beverages like coffee, and especially milk, were more frequently consumed in the high potato consumption group. Median alcohol consumption (g/day) was similar in both groups (Table 2).

**Table 2:** Dietary characteristics of high and low potato consumption in the NOWAC study*

Foods, median g/day†(P5–P95)	Low (*n*=32,493)	High (*n*=41,715)
Potatoes	50 (0–63)	126 (126–189)
Vegetables	127 (32–330)	121 (36–301)
Fish	76 (20–179)	97 (32–218)
Meat	104 (32–199)	107 (39–202)
Pasta/rice	36 (0–94)	22 (0–73)
Bread (whole grain, refined and crisp bread)	110 (31–211)	131 (43–240)
Milk	80 (0–525)	165 (0–600)
Coffee	525 (0–1,470)	735 (0–1,680)
Alcohol	2 (0–13)	2 (0–11)
Fat	59 (33–99)	64 (36–107)
Carbohydrate	175 (98–269)	199 (122–296)
Fibre	20 (10–32)	22 (13–34)
Starch	90 (45–140)	108 (65–157)
Niacin (mg/day)	14 (9–22)	17 (11–24)
Vitamin C (mg/day)	92 (29–225)	93 (40–219)
Folate (µg/day)	168 (93–281)	185 (114–295)
Energy (kJ/day)	6,574 (4,003–9,826)	7,241 (4,634–10,661)

NOWAC, Norwegian Women and Cancer; P5–P95, 5th–95th percentile; Low, ≤1 potato per day; High, ≥2 potatoes per day

* *P*-value<0.001 for all differences; Wilcoxon test

† If not otherwise mentioned.

Potatoes were consumed more frequently outside Oslo, and there was a clear north–south gradient in the consumption: compared to Oslo, people living in the north had the highest odds of being in the high potato consumption group (OR: 4.54, 95% CI: 4.26–4.83) (Table 3). In the mutually-adjusted models the odds were lower, but the trends were still very clear. Women living with children had lower odds of high potato consumption than women living only with a partner (OR: 0.87, 95% CI: 0.84–0.91), but women living alone had the lowest odds of high potato consumption (OR: 0.42, 95% CI: 0.40–0.44). When the variables were mutually adjusted the results were similar, but there was some strengthening of odds for women living alone, and some attenuation of odds for women living with children. Diabetics had lower odds of high potato consumption compared to those without diabetes (OR: 0.73, 95% CI: 0.65–0.82). After mutual adjustment, the associations became even stronger.

**Table 3:** ORs for high consumption of potatoes, according to selected factors: the NOWAC study*

Lifestyle, socioeconomic and health-related variables	Adjusted for sub-cohort OR (95% CI)	All variables are adjusted mutually and for sub-cohort OR (95% CI)
Age (years)		
(Ref. 40–49)		
50–59	1.37 (1.32–1.41)	1.24 (1.19–1.28)
60–70	2.24 (2.12–2.37)	1.80 (1.69–1.92)
*P* for trend	<0.001	<0.001
	Adjusted for age and sub-cohort OR (95% CI)	
Area of residence (ref. Oslo)		
East except Oslo	2.16 (2.04–2.29)	1.75 (1.65–1.86)
South	2.52 (2.31–2.75)	1.98 (1.81–2.16)
West	2.79 (2.62–2.96)	2.24 (2.11–2.39)
Middle	3.20 (2.97–3.45)	2.53 (2.35–2.74)
North	4.54 (4.26–4.83)	3.41 (3.19–3.64)
Income (NOK)		
<150,000	0.94 (0.88–1.00)	1.07 (1.00–1.15)
151,000–300,000	0.87 (0.83–0.90)	0.97 (0.92–1.01)
(ref. 301,000–450,000)		
451,000–600,000	0.74 (0.71–0.78)	0.80 (0.76–0.84)
601,000–750,000	0.52 (0.50–0.55)	0.63 (0.59–0.66)
>750,000	0.42 (0.38–0.45)	0.50 (0.46–0.55)
*P* for trend	<0.001	<0.001
Education (years)		
(ref. ≤9)		
10–12	0.69 (0.66–0.72)	0.79 (0.76–0.83)
≥13	0.44 (0.42–0.46)	0.60 (0.57–0.62)
*P* for trend	<0.001	<0.001
Household structure		
(Ref. living with partner)		
Living alone	0.42 (0.40–0.44)	0.39 (0.37–0.41)
Living with children	0.87 (0.84–0.91)	0.92 (0.88–0.95)
Smoking status		
(Ref. never)		
Former	0.89 (0.85–0.92)	0.85 (0.82–0.88)
Current	1.28 (1.24–1.33)	1.16 (1.11–1.20)
BMI		
(Ref. under/normal)		
Overweight	1.14 (1.10–1.18)	1.06 (1.02–1.09)
Obese	1.12 (1.06–1.18)	1.04 (0.98–1.10)
*P* for trend	<0.001	0.020
Diabetes		
(Ref. non diabetic)		
Diabetic	0.73 (0.65–0.82)	0.66 (0.59–0.74)
Physical activity		
(Ref. low)		
Moderate	1.17 (1.11–1.22)	1.19 (1.14–1.25)
High	1.14 (1.08–1.21)	1.16 (1.09–1.23)
*P* for trend	<0.001	<0.001
	Adjusted for age and sub-cohort OR (95% CI)	As above, but also adjustment for energy intake (kJ)
Dieting†		
(Ref. not dieting)		
Dieting	0.76 (0.72–0.81)	0.70 (0.65–0.75)

NOWAC, Norwegian Women and Cancer; NOK, Norwegian Kroner

* *n*=74,208.

† Information available in a sub-cohort (*n*=22,726).

Women who reported moderate or high physical activity had higher odds of high potato consumption compared to women with low physical activity. Women with a BMI ≥25 had higher odds of high potato consumption than underweight and normal weight women, however in the mutually adjusted model the odds weakened, and the association became non-significant for the obese category.

Current smokers had higher odds of high potato consumption, while former smokers had lower odds. Regarding socioeconomic status, there was a clear trend: the lesser the women's income and education, the higher their odds of high potato consumption (*P* for trend <0.001). Potato consumption also showed a steep age gradient (OR for the age group 60–70 years compared to 40–49 years in the mutually-adjusted model: 1.80, 95% CI: 1.69–1.92). In a sub-cohort of 22,726 women with information on dieting, we found that the women who were dieting had lower odds of high potato consumption compared with those who were not dieting (OR: 0.70, 95% CI: 0.65–0.75).

Regarding different food groups, we found that the higher the consumption of bread, meat, vegetables and fish, the higher the odds of being in the high potato consumption group (Table 4). The largest effect was found for fish (OR: 2.07, 95% CI: 2.01–2.14). When we adjusted for energy intake the odds generally weakened, although the odds for fish were still quite high (OR: 1.62, 95% CI: 1.57–1.67). For meat and vegetables, adjustment for energy intake resulted in lower odds of high potato consumption (OR per 100 g: 0.94, 95% CI: 0.91–0.98, OR: 0.87, CI: 0.86–0.89). The higher consumption of pasta and rice, the lower the odds of high potato consumption, and the effect was still evident after adjusting for energy intake (OR per 25 g: 0.57, CI: 0.56–0.58). With higher consumption of milk and coffee, the odds of high potato consumption were slightly higher. Alcohol consumption was associated with lower odds of high potato consumption.

**Table 4:** OR for high consumption of potatoes by dietary variable: the NOWAC study*

	Adjusted for age and sub-cohort OR (95% CI)	Adjusted for age, sub-cohort and energy intake OR (95% CI)
Bread (per 100 g)	1.78 (1.74–1.83)	1.19 (1.15–1.23)
Fish (per 100 g)	2.07 (2.01–2.14)	1.62 (1.57–1.67)
Meat (per 100 g)	1.42 (1.38–1.47)	0.94 (0.91–0.98)
Vegetables (per 100 g)	1.00 (0.98–1.02)	0.87 (0.86–0.89)
Pasta/rice (per 25 g)	0.67 (0.66–0.68)	0.57 (0.56–0.58)
Milk (per 200 g)	1.19 (1.17–1.20)	1.05 (1.03–1.06)
Coffee (per 200 g)	1.11 (1.10–1.12)	1.10 (1.09–1.11)
Alcohol (per 3 g)	0.89 (0.88–0.89)	0.87 (0.86–0.88)

NOWAC, Norwegian Women and Cancer

* *n*=74,208.

The age-adjusted mean for each nutrient was higher in the high potato consumption group, especially for folate, carbohydrates and proteins (Table 5). However, the age-adjusted nutrient density per 1,000 kJ for each nutrient was quite similar in the low and high consumption groups (Table 5).

**Table 5:** Age-adjusted absolute intake of nutrient and nutrient density in potato consumers: the NOWAC study*

	Age adjusted means (95% CI)		Age adjusted means/1,000 kJ (95% CI)	
	
	Low	High	Low	High
Folate (µg/day)	175.40 (174.78–175.02)	191.99 (191.44–192.54)	26.48 (26.42–26.54)	26.16 (26.11–26.22)
Niacin (mg/day)	14.66 (14.62–14.70)	17.20 (17.16–17.24)	2.22 (2.22–2.23)	2.35 (2.35–2.36)
Vitamin C (mg/day)	104.62 (103.98–105.27)	105.82 (105.26–106.39)	15.84 (15.75–15.92)	14.42 (14.35–14.50)
Fibre (g/day)	20.35 (20.28–20.42)	22.46 (22.40–22.53)	3.07 (3.07–3.08)	3.06 (3.06–3.07)
Starch (g/day)	90.71 (90.39–91.02)	109.25 (108.98–109.53)	13.61 (13.58–13.64)	14.90 (14.88–14.93)
Carbohydrates (g/day)	177.58 (177.01–178.15)	202.97 (202.47–203.48)	26.62 (26.58–26.66)	27.52 (27.49–27.55)
Protein (g/day)	71.02 (70.80–71.24)	79.50 (79.31–79.69)	10.73 (10.71–10.75)	10.83 (10.81–10.84)
Fat (g/day)	61.33 (61.10–61.56)	66.94 (66.74–67.15)	9.12 (9.11–9.14)	8.96 (8.94–8.97)

NOWAC, Norwegian Women and Cancer; Low, ≤1 potato per day; High, ≥2 potatoes per day

* *n*=74,208; *P*-value<0.001 for all differences, except fibre (*P*=0.091), regression analysis (nutrient density).

Tests for interactions did not show any positive results.

## Discussion

The most important findings in our study were the clear north–south gradient in potato consumption, where women living in the north (compared to Oslo) had the highest odds of high potato consumption. Furthermore, the older the women, and the lower their socioeconomic status, the higher were their odds of high potato consumption. Factors like smoking, diabetes and dieting were identified as determinants of potato consumption. Consumption of different foods varied in the low versus the high potato consumption group. The strongest association was found for fish and pasta/rice. Another important finding was that the nutrient densities in the low and high consumption groups were similar. In general, all the studied variables were significantly associated with potato consumption in all models. In a study of this size, however, a statistically significant association is not necessarily related to clinical relevance, and we have only highlighted the factors showing the strongest associations.

The high consumption group consisted of more elderly women, but we also found a steep age-gradient, with higher odds of high potato consumption with increasing age. This result is congruent with other studies (2, 4, 13). It is likely that tradition and trends play an important part in this. Indeed, there has been a drastic decline in potato consumption over the past 40 years, and it is likely that elderly women hold on to this dietary tradition more than younger people do. Also, since increasing age often brings changed marital status, household structure, income, etc., it was important to take these relevant factors into account. In the present study, the higher the women's income and education, the lower their odds of high potato consumption, which is consistent with similar studies (2, 4). Since potatoes are a cheaper alternative to other foods, it is not surprising that income plays a role in potato consumption. Regarding education, one can speculate that women with higher education travel more, experience more of the rest of the world, and therefore are more likely to try foreign or different food.

Concerning household structure, it was not surprising that the women living alone were less likely to be in the high potato consumption group. Even though living alone and having only one income is generally more expensive, it is likely that single people choose to make easier, less time-consuming meals. This seemed to be the case regardless of age, as the effect was seen in age-adjusted models. Other studies have found contradictory results concerning how having children in the household affects potato consumption (2, 4). However in the present study, households with children were less likely to be in the high potato consumption group than households where women were living with a partner. One possible explanation is that children preferred pasta and rice over boiled potatoes. This is in line with our results regarding pasta and rice, as those who ate more pasta and rice had lower odds of high potato consumption, even after adjustment for energy intake. Thus, the larger median consumption (g/day) of pasta and rice in the low potato consumption group was not surprising. These results are also consistent with other studies showing that pasta and rice act as a substitute for boiled potatoes, especially among younger people (2, 13). Another factor regarding lower potato consumption in households with children could be lack of time. In another study participants gave lack of time as the most important reason for low potato consumption (13).

It has been shown that area of residence is an important determinant of potato consumption in Norway (2, 4), and in the present study we found a steep north–south gradient, where the likelihood of high potato consumption increased the farther women in the study lived from Oslo. This is not surprising, as Oslo, the capital of Norway, differs from the rest of the country in many ways. For instance, it is the largest city in the country, and is the region with most immigrants (24). Therefore, it is likely that Oslo works as an entry gate for trends and cultural influences from other parts of the world.

When it comes to health-related factors, we found that diabetics had lower odds of high potato consumption than non-diabetics. The Norwegian Diabetes Association recommends avoiding foods high in carbohydrates that easily raise blood sugar, and potatoes are mentioned as an example rich on those types of carbohydrates (25). Therefore, it is likely that diabetics have a diet with lower potato intake because of the recommendations due to their illness.

Overweight or obese women (BMI≥25) had higher odds of high potato consumption than underweight and normal weight women. In the mutually adjusted model, the trend for this association remained significant, but the odds weakened and the association became non-significant for BMI in the obese category. Women who reported moderate and high physical activity also had higher odds of high potato consumption compared women with low physical activity. Even though the associations with BMI and physical activity were statistically significant, the effect estimates were small, and therefore likely not clinical relevant.

In the sub-cohort of women with information on dieting, results showed that women who were trying to lose weight had lower odds of high potato consumption compared to those who were not dieting. It is not uncommon that women trying to lose weight eat less food, and accordingly eat fewer potatoes. However, since we adjusted for energy intake, the results showed that these women had specifically chosen to cut down on potatoes. It is likely that these women thought of potatoes, and their high glycaemic index/load, as an unwanted food source when dieting.

Smokers had higher odds of high potato consumption, and former smokers had lower odds. Smoking is known to be more common among those with low socioeconomic status, which is congruent with the results of the present study concerning the association between lower income and education and higher odds of high potato consumption. A possible explanation of why former smokers had lower odds of high potato consumption could be that quitting smoking leads to a change in lifestyle, which could also include a change in diet.

Regarding dietary characteristics, we found a considerable difference in energy intake: the median consumption in the high consumption group was almost 700 kJ/day higher than in the low potato consumption group. The median consumption (g/day) of different foods was higher mostly in the high potato consumption group, and it is likely that the higher energy consumption in that group affected the median difference in the other foods. When adjusting for energy intake in logistic regression analyses, some changes in the OR occurred compared to the age-adjusted analyses. The odds weakened for all foods, although the odds for fish were still quite high. This is in line with other studies, in which results showed that those who ate lots of fish also had high potato consumption (2, 26). Fish-eaters have been shown to be highly represented in the north of Norway (26), and it is possible that the high potato consumption we found in the north was connected with the high consumption of fish. However, when we stratified by fish consumption, the differences in potato consumption between north and south were still very clear (data not shown). One study also found that 85% of respondents reported they ate boiled potatoes with meat (2). In the present study, meat-eaters had high odds of being in the high potato consumption group, but they changed to low after adjustment for energy intake. Similar changes happened with vegetables, which was even more surprising considering the view that vegetables are part of a traditional Norwegian supper (2).

It was notable that the median milk consumption was twice as high in the high potato consumption group. It is likely that this association has to do with tradition, and that women eating lots of potatoes have a traditional diet, which includes more milk than women with less traditional diets. The traditional diet could also explain the odds of high potato consumption observed with coffee and bread consumption. However, in our models adjusted for energy intake, the odds of high potato consumption per 200 g of milk and coffee that women drank were not particularly strong. Median alcohol consumption was similar in both groups, but for every 3 g of alcohol women drank, the odds became somewhat lower for high potato consumption.

The median consumption of all the nutrients we included in our analyses was higher in the high potato consumption group, although the difference regarding vitamin C was surprisingly small, considering potatoes are a good source of vitamin C (5). In addition, in the analysis on nutrient density, the mean consumption of vitamin C was actually lower in the high potato consumption group. The nutrient density in the low and high consumption group was quite similar.

The major strength of this study is its large size, and the fact that it is population-based and relatively representative of the Norwegian woman aged 40–70, although women in the NOWAC cohort are on average slightly more educated than women in the general population. In addition, studies performed on the external validity of the NOWAC study, and the validity and reproducibility of the study, did not find any notable sources of selection bias, and the level of reproducibility for the FFQ was within the range reported for similar instruments (19, 21).

One of the weaknesses of this study is that our data are based on self-administered questionnaires. Our findings regarding women with a BMI classified as obese and the higher odds for high potato consumption became non-significant after adjustment for all the other factors. Since the measures of BMI are based on self-reported height and weight among the participants, it is important to keep in mind that some measurement error could have occurred. Several studies on self-reported height, weight and BMI show that the trend was for height to be overestimated and weight and BMI to be underestimated (27). Even though self-reported information is known to be a source of measurement error, this type of error will mostly dilute the associations, and will not likely cause any substantial problems in our study.

Furthermore, our generated variable household structure could be a source of bias. We do not know for sure if women who responded that they lived with more than one person had children in their household, or if they lived with several other adults. However, based on the fact that living in households with several generations is not common nowadays in Norway, it is not likely that this affected the results to a large extent. In addition, we recoded a variable regarding diabetes for which there was a large amount of missing information, which was probably due to the fact that women who didn't have diabetes neglected the question, as it didn't concern them. This recoding could be a source of misclassification error; however due to results from a validation study on self-reported diabetes in the NOWAC study, we considered the recoding of missing values as ‘not having diabetes’ to be reasonable.

The participants included in our study received questionnaires that contained general questions on potato consumption, thus information on preparation methods was not available. However, since boiled potatoes are such a central component of a Norwegian supper, and the typical Norwegian meal pattern only includes one hot meal per day (28), there was no reason to assume that women in our study would differ from this. In addition, in a later, smaller sample from our study, 50% of the women ate boiled potatoes at least once a day, compared to 1% of the women who ate fried potatoes at least once a day.

## Conclusion

The high potato consumption group, on average, consisted of more elderly women, women with lower socioeconomic status, more smokers, and women living with a partner. In addition, there was a clear north–south gradient in potato consumption, where women living in the north had the highest odds of high potato consumption. Women with diabetes had lower odds of high potato consumption compared to non-diabetics. Women on a diet specifically cut down on potato consumption. Furthermore, the high potato consumption group had an especially higher consumption of fish and a lower consumption of pasta/rice. The nutrient density in the low and high potato consumption group was similar. The potato consumption in Norway is high, which makes it important to study the impact of potatoes on health. The results of this study increases our understanding of factors related to potato consumption, and thereby being potential confounding factors in potato–disease associations. We recommend that these factors are considered in future studies of potato consumption and health outcomes.
